# Bilateral Dysfunction of Otolith Pathway in Patients With Unilateral Idiopathic BPPV Detected by ACS-VEMPs

**DOI:** 10.3389/fneur.2022.921133

**Published:** 2022-08-26

**Authors:** Xiaorong Niu, Peng Han, Maoli Duan, Zichen Chen, Juan Hu, Yanfei Chen, Min Xu, Pengyu Ren, Qing Zhang

**Affiliations:** ^1^Department of Otorhinolaryngology Head and Neck Surgery, First Affiliated Hospital of Xi'an Jiaotong University, Xi'an, China; ^2^Department of Otolaryngology Head and Neck Surgery, Department of Clinical Science, Intervention and Technology, Karolinska University Hospital, Karolinska Institute, Stockholm, Sweden; ^3^Department of Otorhinolaryngology Head and Neck Surgery, Ear Institute, Second Affiliated Hospital of Xi'an Jiaotong University, Xi'an, China; ^4^Department of Otorhinolaryngology Head and Neck Surgery, Xinhua Hospital, Shanghai Jiaotong University School of Medicine, Shanghai Jiaotong University School of Medicine Ear Institute, Shanghai Key Laboratory of Translational Medicine on Ear and Nose Diseases, Shanghai, China

**Keywords:** air-conducted sound, benign paroxysmal positional vertigo, cervical vestibular-evoked myogenic potential, ocular vestibular-evoked myogenic potential, vestibular function

## Abstract

**Objective:**

To observe the functional status of the otolith pathway in patients with unilateral idiopathic benign paroxysmal positional vertigo (BPPV) by combining air-conducted sound elicited cervical vestibular-evoked myogenic potential (ACS-cVEMP) and ocular vestibular-evoked myogenic potential (ACS-oVEMP).

**Methods:**

One hundred and eighty patients with BPPV were recruited for conventional cVEMP and oVEMP tests. The abnormal rates of VEMPs were compared between BPPV patients and control participants.

**Results:**

The abnormal rates of cVEMP and oVEMP in BPPV patients were 46.7% (84/180) and 57.2% (103/180) in affected ears, respectively, and 45.0% (81/180) and 56.7% (102/180) in unaffected ears, respectively; both were significantly higher than the abnormal rates of cVEMP and oVEMP in normal control ears. Compared with normal subjects, the cVEMP response rate was lower in affected and unaffected ears in BPPV patients. The abnormal rates of cVEMP and oVEMP were 48.1% (76/158) and 57.6% (91/158) in patients with posterior semicircular canal BPPV, and 36.4% (8/22) and 54.5% (12/22) in lateral semicircular canal BPPV. There was no significant difference in VEMP abnormalities between posterior semicircular canal BPPV and lateral semicircular canal BPPV.

**Conclusion:**

The prevalence of abnormal cVEMPs and oVEMPs in both affected and unaffected ears of patients with BPPV was significantly higher than that observed in the control group. The pathological mechanism of unilateral idiopathic BPPV may be associated with bilateral degeneration of otolith pathways.

## Introduction

Benign paroxysmal positional vertigo (BPPV) is characterized by recurrent spells of vertigo triggered by changes in head position with respect to gravity. BPPV is one of the most common peripheral vestibular disorders leading to vertigo. BPPV is caused by the otoconia being dislodged from the macula into the semicircular canals. Most cases of BPPV are idiopathic in origin and probably result from degeneration of the macula (1).

Vestibular-evoked myogenic potentials (VEMPs) are used as clinical tests of the otolith pathway, including the saccule and utricle. Cervical VEMP (cVEMP) is recorded from the surface of the sternocleidomastoid muscle (SCM) and reflects the function of the saccule and inferior vestibular nerve. Ocular VEMP (oVEMP) is recorded from the skin surface of the inferior oblique muscle and reflects the function of the utricle and the superior vestibular nerve input pathway (2). Patients with BPPV have abnormal VEMPs in affected ears, and the prevalence of abnormal VEMPs is higher in recurrent BPPV (3, 4). A meta-analysis by Chen et al. (5) revealed that several distinctive characteristics of cVEMP tests exist in BPPV patients compared to healthy controls, including longer latency of p13, a lower amplitude of p13-n23, and a higher proportion of absent responses (5). Some researchers have found that oVEMP is more often abnormal in BPPV patients as compared to cVEMP, leading to speculation that utricular dysfunction may be more common than saccular dysfunction (6, 7). Seo et al. (8) found that reduced oVEMP may originate from the partial degeneration of utricular hair cells. Conversely, augmented oVEMP in the affected ears is thought to originate from hypermobility of the stereocilia due to the detachment of otoconia within the utricle. The results of oVEMP do not appear to be related to the recovery from symptoms (8), although some studies report an increase in oVEMP amplitude on the affected side after a successful repositioning procedure, supporting the hypothesis of the return of otoconia into the area of the utricular macula (9, 10). Singh et al. (3) suggested that a large asymmetry ratio is the most potent characteristic of oVEMP in BPPV. In addition, another study found that abnormalities of cVEMP and oVEMP in the unaffected ears of BPPV patients were significantly higher than in the control group (11). Differences in findings between existing studies indicate the need for more studies. Therefore, we used both cVEMP and oVEMP tests elicited by air-conducted sound (ACS) to fully assess the functional status of the otolith pathway in patients with BPPV and aimed to get a more credible result using a larger sample population.

## Materials and Methods

### Patient Selection

Patients with unilateral idiopathic BPPV who visited the Department of Otorhinolaryngology of the Second Affiliated Hospital of Xi'an Jiaotong University from May 2012 to May 2015 were enrolled in this study. We included only patients with their first attack of idiopathic BPPV. The diagnosis of BPPV was based on: (1) a history of episodes of vertigo with changes in head position relative to gravity; (2) vertigo associated with characteristic nystagmus provoked by the Dix-Hallpike test or roll test; (3) absence of identifiable central nervous system disorders that could explain positional vertigo and nystagmus; and (4) absence of any history of neurotological or vestibular disorders, such as Ménière's disease, labyrinthitis, sudden deafness, vestibular neuritis, migraine, and head trauma. Exclusion criteria included: (1) tympanic membrane perforation or conductive/sensorineural hearing loss caused by any outer/middle/inner ear diseases; (2) symptoms of other cranial nerve damage; (3) bilateral onset or multiple semicircular canal involvement; (4) a previous history of craniocerebral trauma, otitis media, or a history of middle or inner ear surgery (12, 13). No patient underwent any form of treatment for BPPV before their participation in this study. Participants underwent a detailed history collection, physical examination, positional tests, and basic hearing tests to exclude other vestibular diseases.

A total of 180 patients (180 affected ears and 180 unaffected ears) were enrolled in the BPPV group, including 65 males and 115 females aged 16–90 years (mean age = 54.38 ± 14.33 years). There were 92 patients affected on the left side (*n* = 180, 51.1%) and 88 patients affected on the right side (*n* = 180, 48.9%); 158 patients had posterior semicircular canal BPPV and 22 patients had lateral semicircular canal BPPV. The details of clinic data are given in Table 1. We also recruited 57 age and gender-matched healthy subjects (114 ears, aged 22–83 years, mean age: 52.37 ± 15.18 years; 21 men) without a history of previous ear disease or dizziness to serve as controls, after confirming normal findings with a neurological examination and pure tone audiogram. All 180 patients and 57 healthy subjects underwent audiometry, ACS-cVEMP, and ACS-oVEMP tests. The study was approved by the regional ethical committee of the Second Affiliated Hospital of Xi'an Jiaotong University. Each participant signed an informed consent form for participation.

**Table 1:** The clinic data of 180 patients with benign paroxysmal positional vertigo (BPPV).

**Age groups**	***n***	**Sex (** ***n*** **)**		**Sides (** ***n*** **)**		**Clinic types (** ***n*** **)**	
		**M**	**F**	**Left**	**Right**	**PSC**	**LSC**
≤ 30	10	5	5	10	0	10	0
31–50	58	19	39	30	28	53	5
51–70	88	30	58	45	43	71	17
>70	24	11	13	7	17	24	0
Total	180	65	115	92	88	158	22

*PSC, posterior semicircular canal; LSC, lateral semicircular canal*.

### cVEMP and oVEMP Recording

To record cVEMP, a supine position was assumed by the subject. There were five electrodes placed on the subject: two active electrodes in the middle of each SCM, two reference electrodes on each sternoclavicular joint, and a ground electrode above the midline of the forehead. Inter-electrode impedance should not exceed 5 kΩ. When receiving tone bursts through an inserted earphone, the subject had to lift her/his head off the pillow in the midline in order to activate the SCM (14). We monitored the background SCM contraction levels, and the instrument only recorded cVEMP responses at a set EMG range of 50–200 μV.

For the recording of oVEMP, a supine position was also assumed. The detailed method of the oVEMP test was laid out in our earlier article (15). Five electrodes were placed on the body surface. Two active electrodes were in line with each pupil and 2 cm below the lower lid margin. Two reference electrodes were below each active electrode at a distance of 1 cm. A ground electrode was located on the midline of the forehead. Inter-electrode impedance should not exceed 5 kΩ. When receiving tone bursts through a calibrated insert earphone, each subject had to gaze upward at a fixed point on the wall with a vertical visual angle of ~30–35°. The recording instrument and the setting of ACS stimulus in the cVEMP and oVEMP tests were the same. The subjects received a 500 Hz short tone burst (rise/fall time = 1 ms; plateau time = 2 ms) through an insert earphone. A GN Otometrics (Taastrup, Denmark) ICS Chartr EP analyzer was used to amplify the electromyographic signal from the stimulated side. The stimulation rate was 5/s. The average response to each of the 50 stimuli was calculated twice and recorded bilaterally. To check whether a VEMP could be elicited in the subject and waveforms could be identified, the default starting intensity of the stimulus was set as 131 dB Sound Pressure Level (SPL). Then stimulus intensity was decreased in steps of 10 dB when VEMPs were present and increased by 5 dB when VEMPs were absent (15).

### Observation Index for cVEMP and oVEMP

The oVEMP and cVEMP were reproducible short-latency biphasic waveforms. A waveform unrecognizable or unrepeatable was regarded as an absent response. The threshold of the VEMP (dB SPL) was the lowest stimulus intensity leading to an identifiable and repeatable biphasic wave. The parameters, such as amplitude (μV) and p1 and n1 latencies (ms) of oVEMP and cVEMP, were measured at a stimulus of 131 dB SPL. The p1 and n1 latencies were measured as the difference between 0 ms and the time of maximal p1 and n1 peaks. The vertical distance between the peaks of p1 and n1 was amplitude.

Normal values in different age groups, including threshold (dB SPL), p1 and n1 latencies (ms), and amplitude (μV) at a stimulus of 131 dB SPL of oVEMP and cVEMP were recorded in our earlier article (15). Normal ranges of those parameters were calculated as mean ± 2 SD in different age groups. Abnormalities were defined as the absence of VEMPs and recorded parameter values outside of the normal ranges.

### Statistical Analysis

Data were analyzed using SPSS Statistics 18.0. The χ^2^ test was used to compare the rate of abnormalities and the response rate between the BPPV group and the normal control group. The rate of abnormalities and the response rate between the affected ears and the unaffected ears of the BPPV group were analyzed with the McNemar test. For continuous variables, parameters, such as thresholds, p1 and n1 latencies, and amplitude, the Kolmogorov–Smirnov test was used to test for normal distribution. Between the BPPV group and the normal control group, the independent-samples *t*-test was used for normal distributions, and the Mann–Whitney U test was used for non-normal distributions. Between the affected ears and unaffected ears of the BPPV group, the paired-samples *t*-test was used for normal distributions, and the Wilcoxon signed-rank test was used for non-normal distributions. The level of statistical significance was set at *p* < 0.05.

## Results

### Comparison of Abnormal Rates of ACS-VEMPs Among the Affected Ears, the Unaffected Ears of BPPV, and Controls

The abnormal rates of cVEMP and oVEMP in the affected ears of 180 patients with BPPV were 46.7% (84/180) and 57.2% (103/180), respectively. In the unaffected ears, abnormal rates for cVEMP and oVEMP were 45.0% (81/180) and 56.7% (102/180), respectively. The abnormal rates of cVEMP and oVEMP were 23.7% (27/114) and 43.0% (49/114) in the 114 normal control ears tested (114 ears of 57 healthy subjects). The details of abnormal parameters of cVEMP/oVEMP in affected, unaffected, and control ears are depicted in Table 2.

**Table 2:** Abnormal parameters of vestibular-evoked myogenic potentials (VEMPs) in affected, unaffected and control ears.

**Groups**	**Abnormal oVEMP (n)**								**Abnormal cVEMP (n)**						
	***n***	**Absent**	**T↑**	**T↑n1↓**	**T↑n1↑**	**T↓**	**T↓A↑**	**n1↑**	***n***	**Absent**	**T↑**	**T↓**	**T↓A↑**	**T↓p1↓**	**p1↑**
Affected ears	103	88	0	1	1	3	4	6	84	75	0	2	0	1	6
Unaffected ears	102	90	1	1	0	0	0	10	81	74	2	2	0	0	3
Control ears	49	45	0	0	0	0	1	3	27	22	0	0	1	0	4

*T: Threshold; n1: n1 latency; A: Amplitude; p1: p1 latency; ↑: increase; ↓: decrease*.

**Figure 1:**
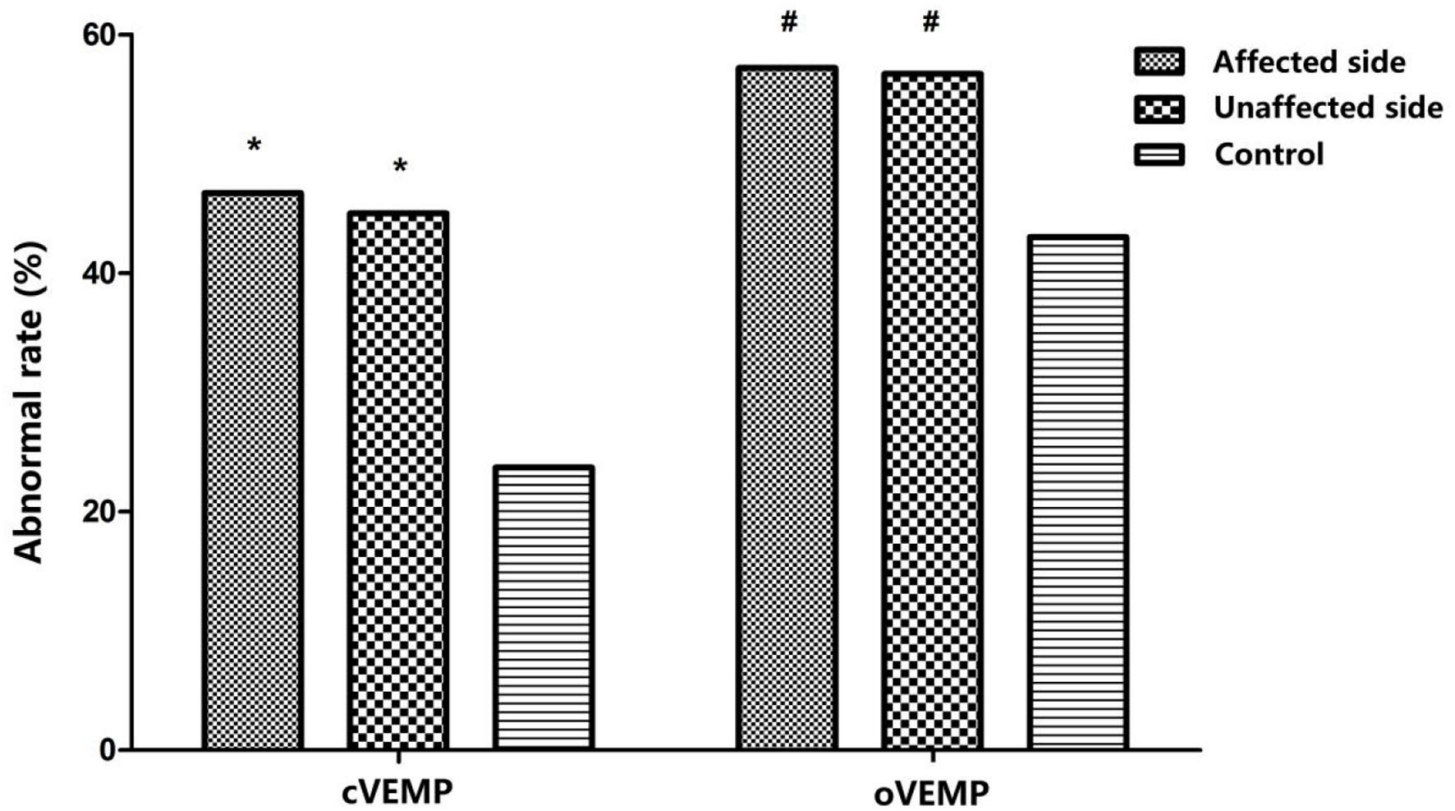
Comparison of abnormal rates of cervical vestibular-evoked myogenic potential (cVEMP) and ocular vestibular-evoked myogenic potential (oVEMP) among affected ears and unaffected ears in the benign paroxysmal positional vertigo (BPPV) group and normal control ears; ^*^p < 0.01, ^#^*p* < 0.05, compared with normal control ears. *p*_affected, cVEMP_ < 0.001, *p*_unaffected, cVEMP_ < 0.001; *p*_affected, oVEMP_ = 0.17, *p*_unaffected, oVEMP_ = 0.22.

Abnormal rates of cVEMP and oVEMP were significantly higher in the affected ears of BPPV patients than those in normal control ears ($\chi_{\text{cVEMP}}^{2}=$ 15.687, *p* < 0.01; $\chi_{\text{oVEMP}}^{2}=$ 5.668, *p* < 0.05). The unaffected ears of BPPV patients similarly had abnormal rates of cVEMP and oVEMP, higher than those in normal control ears (χ^2^_cVEMP_= 13.646, *p* < 0.01; $\chi_{\text{oVEMP}}^{2}$ = 5.232, *p* < 0.01) (Table 3; Figure 1). The abnormal rates of cVEMP or oVEMP between the affected ears and unaffected ears in patients with BPPV did not differ (McNemar test, *p* > 0.05). The results suggest that the abnormal rates of cVEMP and oVEMP are significantly increased bilaterally in patients with BPPV.

**Table 3:** Comparison of abnormal rates of cervical vestibular-evoked myogenic potential (cVEMP) and ocular vestibular-evoked myogenic potential (oVEMP) among different groups.

**Groups**	**Ears (** ***n*** **)**		
	**Affected (180)**	**Unaffected (180)**	**Controls (114)**
cVEMP	46.7% (84/180)*	45.0% (81/180)*	23.7% (27/114)
oVEMP	57.2% (103/180)^#^	56.7% (102/180)^#^	43.0% (49/114)

*p < 0.01, ^#^p < 0.05, Compared with the normal controls, χ^2^ test.

### Comparison of Parameters of ACS-VEMPs Among the Affected Ears, Unaffected Ears of BPPV, and Controls

Among 180 patients with BPPV, cVEMP was recorded in 105 (58.3%) affected ears of BPPV patients (aged 16–79 years, mean age = 50.77 ± 13.95 years), and in 106 (58.9%) unaffected ears (aged 16–84 years, mea*n* = 50.86 ± 13.60 years), respectively. Among 114 normal control ears, cVEMP was recorded in 92 (80.7%) ears (aged 22–83 years, mea*n* = 50.42 ± 15.43 years). The average parameters of each group, such as thresholds, p1 and n1 latencies, inter-peak latency, and amplitude, are shown in Table 4.

**Table 4:** Comparison of cervical vestibular-evoked myogenic potential (cVEMP) parameters among affected, unaffected, and control ears.

**Groups**	**Ears**		
	**Affected**	**Unaffected**	**Controls**
Response rate	58.3%*	58.9%*	80.7%
	(105/180)	(106/180)	(92/114)
p1 latency (ms)	16.22 ± 2.49	16.09 ± 2.35	16.70 ± 2.20
n1 latency (ms)	23.41 ± 2.52	23.37 ± 2.61	23.79 ± 2.28
p1-n1 interval (ms)	7.15 ± 1.83	7.32 ± 1.83	7.09 ± 1.78
Amplitude (μV)	138.13 ± 83.56	151.89 ± 98.61	152.10 ± 78.69
Threshold (dB SPL)	123.19 ± 5.76	123.26 ± 5.94	122.36 ± 5.65

^*^Compared with the normal controls, p < 0.01, χ^2^test.

**Figure 2:**
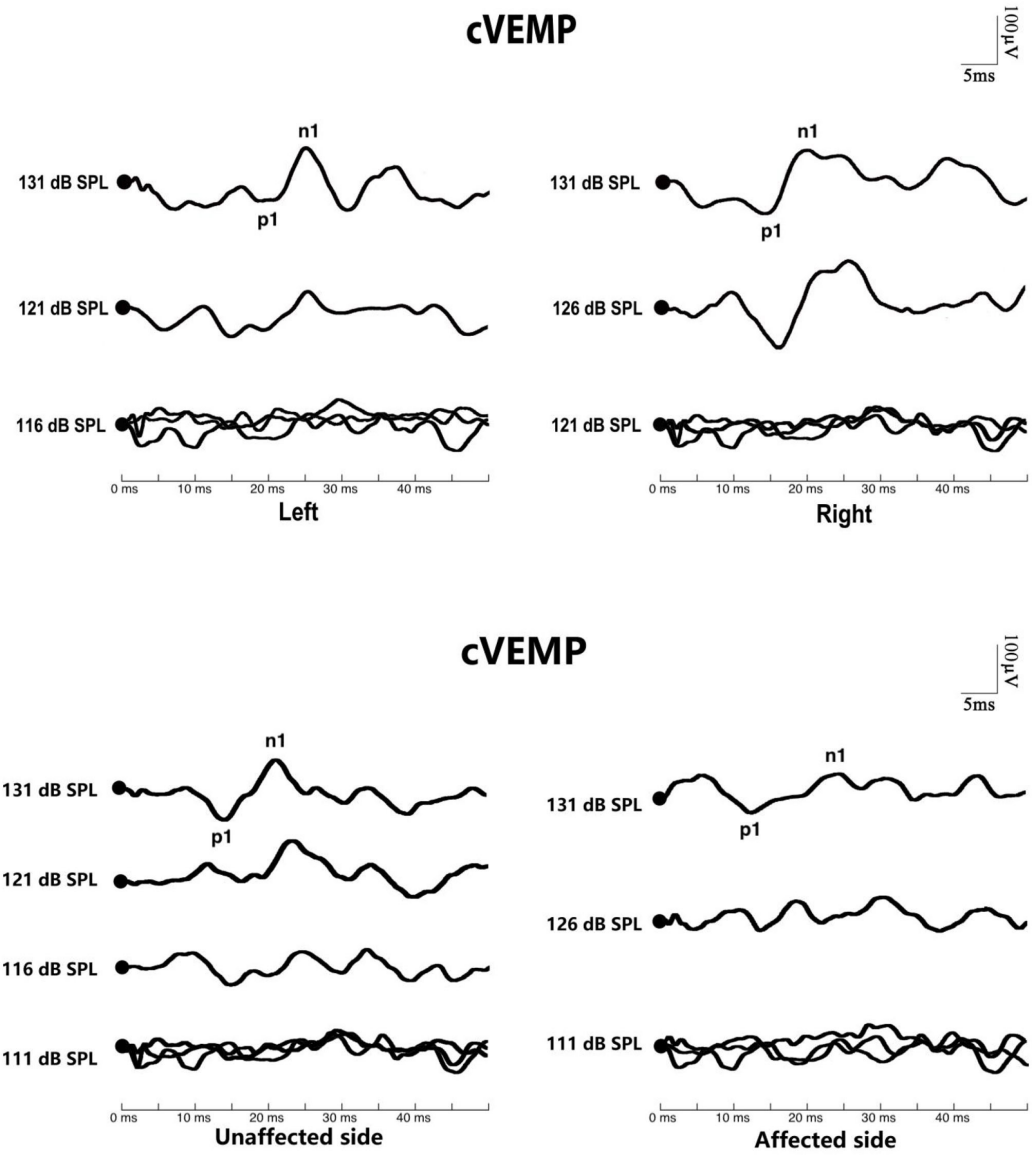
Cervical vestibular-evoked myogenic potential (cVEMP) waves of patients with benign paroxysmal positional vertigo (BPPV). The top picture shows the cVEMP response of a normal subject. The bottom picture shows the cVEMP response of a patient with BPPV.

The response rate of cVEMP in the affected ears of BPPV patients (χ^2^= 15.796, *p* < 0.01) and that in the unaffected ears of BPPV patients (χ^2^= 15.101, *p* < 0.01) were significantly lower than that in normal control ears. The affected and unaffected ears of BPPV patients did not differ in cVEMP response rate (McNemar test, *p* > 0.05). There were no significant differences in cVEMP threshold, p1 latency, n1 latency, or amplitude in affected/unaffected ears of BPPV patients vs. normal control ears (*p* > 0.05, independent-samples *t-*test), and in the affected vs. unaffected ears of BPPV patients (*p* > 0.05, paired-samples *t-*test) (Table 4; Figure 2).

Parameters of oVEMP were similarly compared in affected and unaffected ears of BPPV patients and normal control ears. Among 180 BPPV patients, an oVEMP was recorded in 92 (51.1%) affected ears of BPPV patients (aged 16–77 years, mea*n* = 49.24 ± 13.91 years), and in 90 (50.0%) unaffected ears of BPPV patients (aged 16–74 years, mea*n* = 47.72 ± 13.13 years), respectively. Among the 114 normal control ears, oVEMP was recorded in 69 (60.5%) cases (aged 22–83 years, mea*n* = 46.59 ± 14.50 years). The average parameters of each group, such as thresholds, p1 and n1 latencies, inter-peak latency, and amplitude, are shown in Table 5.

**Table 5:** Comparison of ocular vestibular-evoked myogenic potential (oVEMP) parameters among affected, unaffected, and control ears.

**Groups**	**Ears**		
	**Affected**	**Unaffected**	**Controls**
Response rate	51.1%	50.0%	60.5%
	(92/180)	(90/180)	(69/114)
n1 latency (ms)	10.34 ± 0.88	10.44 ± 1.06	10.51 ± 0.76
p1 latency (ms)	14.96 ± 1.67	15.11 ± 1.66	14.89 ± 1.48
p1-n1 interval (ms)	4.62 ± 1.37	4.66 ± 1.31	4.38 ± 1.32
Amplitude (μV)	5.46 ± 4.21	4.91 ± 3.46	5.01 ± 3.27
Threshold (dB SPL)	123.93 ± 5.65	124.11 ± 4.83	123.03 ± 5.31

**Figure 3:**
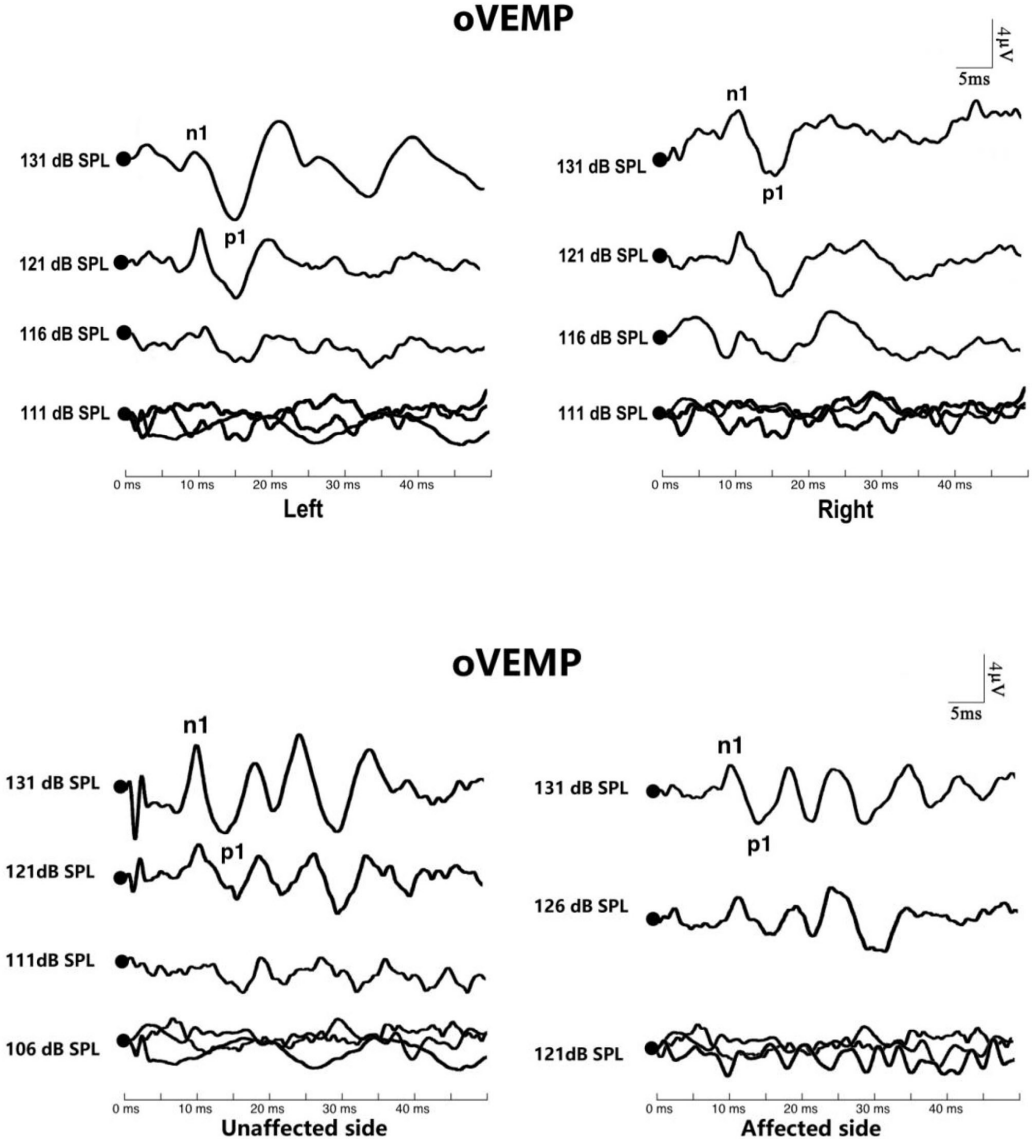
Ocular vestibular-evoked myogenic potential (oVEMP) in affected ears with benign paroxysmal positional vertigo (BPPV). The top picture shows the oVEMP response of a normal subject. The bottom picture shows the oVEMP response of a patient with BPPV.

The response rate of oVEMP did not differ in the affected/unaffected ears of BPPV patients *vs*. normal control ears (χ^2^ test, *p* > 0.05), and in the affected ears vs. unaffected ears in patients with BPPV (McNemar test, *p* > 0.05). There were no significant differences in oVEMP threshold, p1 latency, n1 latency, or amplitude between the affected/unaffected ears of BPPV patients and normal control ears (*p* > 0.05, independent-samples *t-*test), and between the affected ears and unaffected ears in patients with BPPV (*p* > 0.05, paired-samples *t-*test), respectively (Table 5; Figure 3).

### Comparison of Abnormal Rates of ACS-VEMPs Between Different Types of BPPV

Among 180 patients with unilateral BPPV, there were 158 cases of posterior semicircular canal BPPV (age range = 16–90 years, mean age = 54.54 ± 14.94 years). In this group, the abnormal rate of cVEMP was 48.1% (76/158), and the abnormal rate of oVEMP was 57.6% (91/158). The remaining 22 cases were of lateral semicircular canal BPPV (age range = 34–69 years, mean age = 53.27 ± 8.89 years). For this group, the abnormal rate of cVEMP was 36.4% (8/22), and the abnormal rate of oVEMP was 54.5% (12/22). A χ^2^ test was performed, and abnormal rates of cVEMP and oVEMP did not differ significantly between the posterior semicircular canal BPPV group and the lateral semicircular canal BPPV group ($\chi_{\text{cVEMP}}^{2}$ = 1.069, *p* > 0.05; $\chi_{\text{oVEMP}}^{2}$ = 0.073, *p* > 0.05) (Table 6).

**Table 6:** Comparison of abnormal rates of vestibular-evoked myogenic potentials (VEMPs) between two types of benign paroxysmal positional vertigo (BPPV).

**Groups**	**Semicircular canal BPPV (** ***n*** **)**	
	**Posterior (158)**	**Horizontal (22)**
cVEMP	48.1% (76/158)	36.4% (8/22)
oVEMP	57.6% (91/158)	54.5% (12/22)

## Discussion

From 17 to 42% of patients with vertigo ultimately were diagnosed with BPPV, making BPPV the most frequent peripheral system cause of vertigo, in general, and the most common cause of vestibular organ-related vertigo, in particular (16, 17). One cross-sectional research from Europe reported a 2.4% lifetime prevalence of BPPV and 10.7 to 64 cases per 100,000 per year incidence (18, 19). The peak incidence of the disorder occurs between the fifth and sixth decades of life. Women are disproportionately affected, with a ratio of 2–3 to 1 compared to men (17, 18). Among the three semicircular canals, the posterior canal variant is the most common type of BPPV (80–90%). Horizontal canal and superior canal BPPV represent only 10–20% and 3% of cases, respectively (20–22). To avoid confounding factors from multicanal and bilateral cases, our study included only patients with BPPV present in a single semicircular canal. The age distribution, sex ratio and proportion of clinical types of all included BPPV patients were similar to those in previous reports.

Earlier histopathologic research by Dix and Hallpike (23) and Schuknecht (24) suggested that BPPV may be caused by the displacement of utricular otoconia. Then, Moriarty et al. (25) and Naganuma et al. (26) found the basophilic cupular deposits in human temporal bones. Later, a study of 186 temporal bones aged from newborn to 10 years showed that the occurrence of the basophilic cupular deposits, which have been clinically associated with BPPV, was lower in children. Also, the granular particles in basophilic deposits were indistinguishable from otoconia observed on the otoconial membrane of the utricular macula. They speculated that aging of the vestibular labyrinth could be the cause of the accumulation of deposits. In recent work by Kao et al. (27), a scanning electron micrograph of the posterior semicircular canal from patients with intractable BPPV found free-floating otoconia linked to a gelatinous matrix by linking filaments. The free-floating particles leading to BPPV are thought to be otoconia dislodged from the utricle (1, 28). BPPV is typically caused by degeneration of the utricular macula and is idiopathic in origin (1). VEMPs are routinely used as clinical tests of saccule and utricle function. VEMPs abnormalities in BPPV have been reported in several pieces of research, and the prevalence was higher in recurrent BPPV (7, 29–31).

Ocular vestibular-evoked myogenic potential (oVEMP) responses were reported as having more association with BPPV than cVEMP, the interpretation being that the utricle is considered to play a more important role than the saccule, because of the anatomical relation with the semicircular canals (SCCs) (7). Nakahara et al. (7) found abnormal oVEMPs and cVEMPs in 66.7% and 16.7% of posterior SCC BPPV patients, respectively. They considered that the higher incidence of abnormal oVEMPs suggested dislodgement of otoconia from the utricular macula led to utricular dysfunction (7). Kim et al. (11) showed that the abnormal rates of both cVEMP and oVEMP were significantly higher in patients with BPPV than in healthy controls. Our results that show the proportions of abnormal cVEMP and oVEMP in the affected ears of BPPV patients were significantly higher than that in normal control ears are consistent with those of Kim et al. (11) and indicate that BPPV patients have decreased function of both saccular and utricular pathways compared with normal healthy individuals.

At the same time, we found no significant difference between affected ears and unaffected ears in the BPPV group for abnormal rates of cVEMP or oVEMP. However, when compared to the control group, unaffected ears in the BPPV group showed significantly higher abnormal rates of cVEMP and oVEMP. Similar results have been reported by other investigators. For example, Nakahara et al. (7) found that 75% of patients with abnormal oVEMPs showed bilaterally abnormal responses. Kim et al. (11) reported significantly higher abnormal rates of cVEMP and oVEMP on both the unaffected and affected sides of BPPV patients. This suggests that even when BPPV patients have only unilateral clinical symptoms, dysfunction of saccular and utricular pathways may occur bilaterally. BPPV is considered to be caused by the detachment of otoconia from the macula of the utricle. However, increasing evidence suggests that both the utricle and the saccule could be affected by the macular degenerative process (32–36). Although the pathology of the detachment of the otoconia is not yet fully understood, it was thought that a decrease in the gelatinous layer of the otolithic membrane could be caused by degenerative changes, and that may lead to spontaneous dislodgment of the otoconia from the utricular or saccular macula (30, 37). With aging, the otoconia get pitted, fissured, penetrated, and eventually broken into fragments (38). Osteopenia, osteoporosis, and vitamin D deficiency may also cause deranged calcium metabolism in the vestibular organs and may be related to the incidence of BPPV (39). Thus, it appears that macular degenerative changes, caused by increasing age or abnormal calcium metabolism, lead to BPPV and that this contributes to the development of bilateral otolith dysfunction. When this occurs, the otoconia can fall off the macula on either side and enter the semicircular canal, causing clinical symptoms on the corresponding side.

Abnormalities in VEMPs in this study included the absence of a waveform and recorded parameter values outside the normal range for the age group. The response rates of cVEMPs in the affected and unaffected ears of patients with BPPV were significantly lower than in normal control ears. The response rate of oVEMP did not differ in the affected/unaffected ears of BPPV patients *vs*. normal control ears. This may be related to the low response of ACS-oVEMP being affected by age. The waveform of oVEMP was elicited in only 60.5% of normal control ears. There were no significant differences in oVEMP threshold, p1 latency, n1 latency, or amplitude between the affected/unaffected ears of BPPV patients and normal control ears, and between the affected ears and unaffected ears in patients with BPPV, respectively. The abnormalities of VEMPs elicited by ACS in patients with BPPV presented mainly as an absence of response. The recorded waveform of BPPV patients was similar to that of normal control ears. Different results have been reported in the past in this regard. Some studies have found that the waveform parameters of VEMPs in patients with BPPV have prolonged latency, amplitude reduction, and abnormal asymmetry ratio (AR) value (3, 4). Similar to our study, Singh et al. (40) found that the latency or amplitude did not differ between healthy individuals and those with BPPV.

Yetiser et al. (29) and Singh et al. (40) noted that the abnormality of cVEMP is not related to which semicircular canal is affected in BPPV patients. Kim et al. (11) compared the abnormal rates of cVEMP and oVEMP between 47 patients with posterior semicircular canal BPPV and 51 patients with lateral semicircular canal BPPV and found no significant differences (11). In the current study, the abnormal rates of cVEMPs and oVEMPs similarly did not differ between different types of BPPV.

Gacek (34) proposed that BPPV may result from a morphological change associated with the aging labyrinth that leads to cupular deposits. He proposed that the inhibitory action of otolith organs to canal activation was lost due to the degeneration of otolith neurons and that this explains the brief canal response induced by the positional stimulus. He further proposed that the inadequate inhibition in the saccular macula may lead to nystagmus of posterior canal BPPV, while decreased utricular inhibition of lateral canal activation may be presented in lateral canal BPPV (34). We speculate that this concept may offer a better explanation for patients with chronic BPPV, refractory BPPV, and subjective BPPV. In contrast, our findings do not support the above hypotheses.

## Conclusion

The occurrence of abnormal cVEMP or oVEMP in both affected and unaffected ears of patients with BPPV was significantly higher than that observed in the ears of the control group, indicating that otolith pathway dysfunction occurs bilaterally and simultaneously in both saccular and utricular pathways. Thus, the pathological mechanism of unilateral idiopathic BPPV may be associated with bilateral degeneration of otolith pathways. The affected side of those with BPPV that creates symptoms may occur at random or may be related to physical factors, such as sleeping position and anatomical structure of the inner ear.

## Limitations

The ACS-oVEMP we used may be more sensitive than BCV-oVEMP in detecting disease but is associated with more false-positive (abnormal) results. We also have a portion of older subjects, though control subjects were not significantly different in age compared with BPPV subjects. Furthermore, ACS VEMPS can be affected by even minor air-bone gaps. It must be admitted that we should be cautious in interpreting absent ACS-oVEMPs, especially in patients with hearing loss and elderly patients. Therefore, although we have the preliminary results above, oVEMP and whether specific differences arise from the use of BCV or ACS stimulation could be the focus of further studies.

## Data Availability Statement

The raw data supporting the conclusions of this article will be made available by the authors, without undue reservation.

## Ethics Statement

The studies involving human participants were reviewed and approved by Regional Ethical Committee of the Second Affiliated Hospital of Xi'an Jiaotong University. Written informed consent to participate in this study was provided by the participants' legal guardian/next of kin.

## Author Contributions

XN and PH wrote the manuscript. PR, MX, and QZ provided the idea for the study and reviewed and edited the manuscript. MD reviewed and edited the manuscript. ZC, YC, and JH collected the clinical data. All authors contributed to the article and approved the submitted version.

## Funding

This study was funded by the Natural Science Foundation of China (Nos. 81803317, 81970891, and 82171137), Natural Science Foundation of Shaanxi Province (No. 2019JQ-957), The Key International Cooperation Project of Shaanxi Province (No. 2020-KWZ-019), and Fundamental Research Funds for the Central Universities, China (No. xjj2018094).

## Conflict of Interest

The authors declare that the research was conducted in the absence of any commercial or financial relationships that could be construed as a potential conflict of interest.

## Publisher's Note

All claims expressed in this article are solely those of the authors and do not necessarily represent those of their affiliated organizations, or those of the publisher, the editors and the reviewers. Any product that may be evaluated in this article, or claim that may be made by its manufacturer, is not guaranteed or endorsed by the publisher.

## Abbreviations

BPPV: benign paroxysmal positional vertigo
VEMP: vestibular evoked myogenic potential
SCM: sternocleidomastoid
cVEMP: cervical VEMP
oVEMP: ocular VEMP
ACS: air-conducted sound
BCV: bone-conducted vibration
EMG: electromyogram.
